# The evaluation of levosimendan in patients with acute myocardial infarction related ventricular septal rupture undergoing cardiac surgery: a prospective observational cohort study with propensity score analysis

**DOI:** 10.1186/s12871-022-01663-z

**Published:** 2022-05-03

**Authors:** Ze-Shi Li, Kuo Wang, Tuo Pan, Yan-Hua Sun, Chang Liu, Yong-Qing Cheng, He Zhang, Hai-Tao Zhang, Dong-Jin Wang, Zu-Jun Chen

**Affiliations:** 1 Department of Cardio-Thoracic Surgery, Nanjing Drum Tower Hospital, Peking Union Medical College & Chinese Academy of Medical Sciences, Graduate School of Peking Union Medical College, Nanjing, 210008 Jiangsu China; 2 Department of Cardio-Thoracic Surgery, Nanjing Drum Tower Hospital, XuZhou Medical University, Nanjing, Jiangsu China; 3 Department of Anesthesia, Nanjing Drum Tower Hospital, The Affiliated Hospital of Nanjing University Medical School, Nanjing, Jiangsu China; 4 Department of Cardio-Thoracic Surgery, Nanjing Drum Tower Hospital, The Affiliated Hospital of Nanjing University Medical School, Nanjing, Jiangsu China; 5 The Department of Intensive Care Unite, Chinese Academy of Medical sciences & Peking Union Medical College, Fuwai Hospital, Beijing, China

**Keywords:** Levosimendan, Ventricular septal rupture, Cardiac surgery, Coronary artery bypass grafting, Mortality, Postoperative complication

## Abstract

**Study objective:**

The purpose of the present study was to evaluate the efficacy of levosimendan in patients with acute myocardial infarction related ventricular septal rupture (AMI-VSR) underwent cardiac surgery.

**Design:**

Prospective observational cohort study with propensity score analysis.

**Patients:**

There were 261 patients with AMI-VSR in our study. After 1:1 propensity matching, 106 patients (53 levosimendan and 53 control) were selected in the matched cohort.

**Interventions:**

None.

**Measurements:**

Patients who received levosimendan were assigned to the levosimendan group (*n* = 164). The patients who were not received were levosimendan assigned to the control group (*n* = 97). The levosimendan was initiated immediately after cardiopulmonary bypass. Then, it has been maintained during the postoperative 3 days. The poor outcomes were identified as follows: death and postoperative complications (postoperative stroke, low cardiac output syndromeneeded mechanical circulatory support after surgery, acute kidney injury (≥ stage III), postoperative infection or septic shock, new developed atrial fibrillation or ventricular arrhythmias).

**Main results:**

Before matching, the control group had more length of ICU stay (6.69 ± 3.90 d vs. 5.20 ± 2.24 d, *p* < 0.001) and longer mechanical ventilation time (23 h, IQR: 16–53 h vs. 16 h, IQR: 11–23 h, *p* < 0.001). Other postoperative outcomes have not shown significant differences between two groups. After matching, no significant difference was found between both groups for all postoperative outcomes. The Kaplan–Meier survivul estimate and log-rank test showed that the 90-day survival had no significant differences between two groups before and after matching.

**Conclusion:**

Our study found that a low-dose infusion of levosimendan in AMI-VSR patients underwent surgical repair did not associated with positively affect to postoperative outcomes.

## Introduction

Ventricular septal rupture (VSR) is a fatal complication of acute myocardial infarction (AMI) [1]. The mortality among VSR patients is nearly 41–80% [2, 3]. Surgical repair may be the best choice for VSR compared with other treatments [4]. However, it had been reported that the mortality of surgical repair was from 38.2 to 65% [4–6], it is the highest mortality among all kinds of cardiac surgery. Meanwhile, the surgical repair may have some severe postoperative complications which related to poor outcome, such as acute kidney injury (AKI), low cardiac output syndrome (LOCS) and hepatic failure [6, 7]. Thus, preserving hemodynamic stabilization is necessary and crucial. Inotropic agents and mechanical circulatory support (MCS) devices (intra-aortic balloon pump and extracorporeal membrane oxygenation) were usually administrated for hemodynamic stabilization in these patients. However, the traditional inotropic agents have adverse effects in patients with severe left ventricular dysfunction and coronary vascular disease [8]. And the MCS could cause some life-threatening complications [9]. In addition, recent studies reported that the MCS might not reduce short- or long-term mortality [10, 11]. Thus, some novel inotropic agents might be need to develop for patients with VSR.

Levosimendan is a calcium sensitizer that exerts its inotropic effect by interacting with troponin C (the binding protein for calcium) to enhance the calcium sensitivity of cardiac myocytes [12]. Therefore, levosimendan can improve cardiac performance while not increasing myocardial oxygen consumption or changing myocardial substrate utilization [12]. Some previous studies had shown that levosimendan improved cardiac function in high-risk patients underwent cardiac surgery [13, 14]. It could significantly decreased mortality and postoperative complications [15–17]. However, some trials found that levosimendan might have no benefit in patients undergoing cardiac surgery [18–20]. In a word, the efficacy of levosimendan in patients undergoing cardiac surgery is still controversial. Moreover, there is no study that has been developed to evaluated levosimendan in patients with VSR. Thus, we designed this prospective observational cohort study to evaluate the efficacy of levosimendan in patients with VSR underwent cardiac surgery.

## Materials and methods

### Study design and patients

The VSR is a rare complication of AMI. It was not easy to get an accepted sample size in a single-center random control trial (RCT). Therefore, we designed a prospective observational cohort in two medical centers. This study included all those patients in two tertiary hospitals with VSR and undergoing cardiac surgery from January 1, 2015 to October 1, 2021. According to the ethical guidelines of the Helsinki declaration, ethical committees of Nanjing Drum Tower Hospital and Chinese Academy of Medical Sciences Fuwai Hospital had approved the study. The written informed consents were obtained from the patients or a member of their authorized delegator. Whether to use levosimendan was based on the treating physician’s treatment strategy.

All patients who developed VSR diagnosed by echocardiography with low ejection fraction (≤35%) and scheduled for concomitant coronary artery bypass grafting (CABG) with cardiopulmonary bypass (CPB) were included. The exclusion criteria were as follows: 1) The patients who died before surgery; 2) post-AMI VSR with free wall rupture or papillary muscle dysfunction [21]; 3) urgent operation; 4) re-operation for postoperative residual shunt and tearing attributed to sutures poorly; 5) preoperative MCS used; 6) Other medical diseases or conditions (i.e. cancers, pregnant, lactation period, autoimmune disease,multi-organ failure, ongoing infection).

### Study treatment protocol

Before operation, Swan-Ganz catheter was placed routinely. Durgs and dosage used during anesthesia were as described below. For inducing general anesthesia, Vecuronium: 0.07–0.15 mg/kg, Etomidate: 0.1–0.4 mg/kg, midazolam: 0.1–0.4 mg/kg, and sufentanil: 0.5–1.0 μg/kg were used. In continuous anesthesia, sevoflurane mixed with oxygen (< 4.0%), 25–75 mg/kg/min of propofol,: 0.1–0.2 mg/kg/min of remifentanil and 1.0–2.0 mg/kg/min of vecuronium were used. Intermittent positive pressure breathing (IPPB) for provide intraoperative intermittent mechanical ventilation. Tidal volume was 6–10 mL/kg, fraction of inspired oxygen (FiO2) was 0.6–1.0 and 4–7 cm H_2_O of positive end expiratory pressure (PEEP). β-blocker and amiodarone were used for control arrhythmia and/or tachycardia after excluded contraindications.

All operations were performed with standard CPB. Arterial cannulation used an appropriate size cannula inserted into the ascending aorta, single-stage cannulae with superior and inferior vena cavae or dual-stage cannulae with right atrium were chosen for venous cannulations. The CPB circuit priming with 1500–2000 mL Sodium Lactate Ringer’s Injection contained 25–50 g albumin and 20 mL 10% Magnesium Sulfate Injection. Intravenous infusion 200–400 U/kg of heparin for anticoagulation, and CPB was started when whole-blood active clotting time (ACT) was over 480 s. Antegrade cardioplegia used hyperkalemic cold blood cardioplegia (cardioplegia solution to blood ratio was 4:1), which was delivered every 20 to 30 min through the aortic root during the aortic cross-clamp (ACC). At the end of CPB, 1:1 ratio of protamine for reverse heparin. The CABG was done before VSR repair. Two surgical techniques were used for VSR repair (Daggett procedure and David procedures), and determined by the cardiac surgeons. The Dagget procedure used single or multiple patches to cover the defect and sewed to the LV and RV to close the VSR [22]. The David procedure placed all sutures in the LV, which is also named “infarct exclusion technique” [23].

For patients in the levosimendan group, intravenous infusion of levosimendan was initiated immediately after CPB. The levosimendan was administrated as follows: loading dose was 6 μg/kg in the first hour, followed by a maintenance dose of 0.1 μg /kg/min. The infusion of levosimendan was maintained during the postoperative 3 days if clinically appropriate. Other inotropes and vasopressors were routinely used. For patients in control groups, the treatment strategy was the same excepted levosimendan.

### Definition

Postoperative complications were the following in-hospital postoperative complications included postoperative stroke, low cardiac output syndrome (LCOS) needed mechanical circulatory support (MCS) after surgery, postoperative acute kidney injury (≥ stage III), postoperative infection or septic shock, new devoleped atrial fibrillation or ventricular arrhythmias. Diagnosis and classification of AKI was based on KDIGO clinical practice guideline [24].

Vasoactive inotropic score (VIS) was used for quantifies the amount of cardiovascular support required by patients postoperatively and includes dopamine, dobutamine, epinephrine, milrinone, vasopressin, and norepinephrine. VIS was calculated (VIS = dopamine dose [μg kg − 1 min − 1] + dobutamine [μg kg − 1 min − 1] + 100 × epinephrine dose [μg kg − 1 min − 1] + 10 × milrinone dose [μg kg − 1 min − 1] + 10,000 × vasopressin [units kg − 1 min − 1] + 100 × norepinephrine dose [μg kg − 1 min − 1]). VISmax defined as using the maximum dosing rates of vasoactive and inotropic medications (μg kg − 1 min − 1) during the first 24, 48, 72 h after postoperative ICU admission.

### Statistical analysis

IBM SPSS Statistics (version 26; IBM Corporation, Armonk, NY) was used for analysis. Continuous variables were described as mean ± SD or median with interquartile ranges (IQR). Discrete variables were described as frequencies (n, %). continuous normally distributed variables were compared by independent samples Student’s t test, Mann-Whitney U test were used to compare variables not normally distributed. The categorical variables were compared using Chi-square or Fisher exact test when appropriate. A *P* value < 0.05 was considered as significant.

Further assessment used propensity score analysis for adjustment indicated bias and keep the homogeneity comparable between two groups.

We selected age, EuroSCORE, NYHA class, hypertension, LVEF, ACC and CPB time for including in matching. They were proven to be related with poor outcomes after cardiac surgery [25–28]. For each patient, the probability of administrating levosimendan and compared with patients who had not in a 1:1 ratio, matched by the closest propensity score with ±0. 01 difference. Standardized Mean Difference (SMD) was used for the assessment of balance after match. Then, we used Kaplan–Meier survival estimate to contrast the postoperative 90- day survival of two groups befor and after matching.

## Results

### Clinical characteristics

**Fig. 1:**
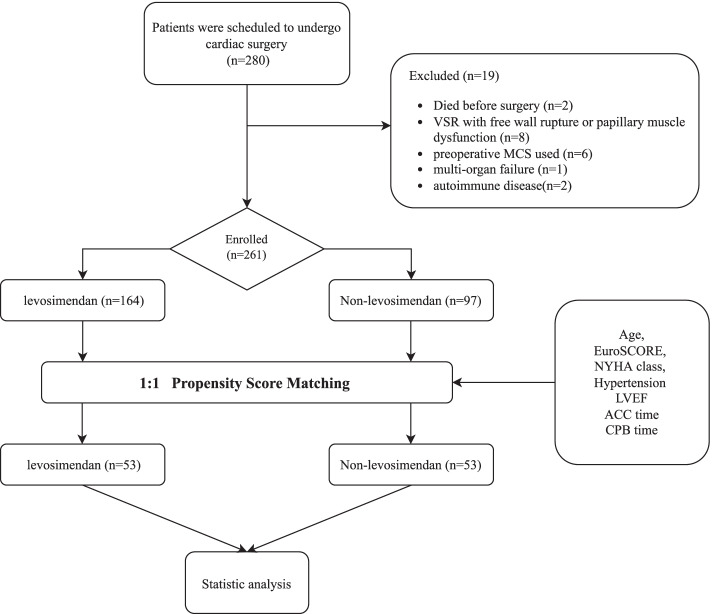
Consort diagram of patient screening and allocation

From January 1, 2015 to September 1, 2021, the study enrolled 280 patients totally, and 261 patients were included in the statistic analysis and were followed for 90 days after surgery (Fig. 1).

In 261 patients, 164 patients used levosimendan, 97 patients were not. The demographic data and preoperative clinical characteristics have no difference between two groups (Table 1). Chinese Academy of Medical Sciences Fuwai Hospital enrolled 241 patients and mortality was 32.37%, Nanjing Drum Tower Hospital enrolled 20 patients and mortality was 35.00%, There was no significant difference in mortality between two centers (*P* = 0.739).

**Table 1:** Baseline and characteristics

Variable	Pre-match			Propensity-match		
	Control (***n*** = 97)	Levosimendon (*n* = 164)	***P*** value	Control (***n*** = 53)	Levosimendon (*n* = 53)	***P*** value
Age (year)	57.19 ± 11.84	58.17 ± 12.03	0.521	58.91 ± 11.67	60.19 ± 13.07	0.595
Gender (male, %)	54 (55.67)	110 (67.07)	0.681	30 (56.60)	30 (56.60)	–
Body mass index (kg/m^2^)	24.23 ± 4.48	24.06 ± 4.63	0.793	24.53 ± 4.41	24.23 ± 4.95	0.770
NYHA class (III /IV)	39/58	50/114	0.137	13/40	15/38	0.826
EuroSCORE (%)	14.78 ± 5.85	15.59 ± 3.55	0.223	14.85 ± 6.20	15.28 ± 3.93	0.668
Medical History						
Myocardial infarction ≤7 d (n,%)	49 (50.52)	83 (50.61)	0.988	30 (56.60)	26 (49.06)	0.560
Myocardial infarction ≤30 (n,%)	97 (100)	164 (100)	–	53 (100.00)	53 (100.00)	–
Atrial fibrillation(n,%)	38 (39.18)	69 (42.07)	0.697	19 (35.85)	21 (39.62)	0.841
Chronic Renal Failure (n,%)	21 (21.65)	28 (17.07)	0.413	10 (18.87)	9 (16.98)	0.988
Dyslipidemia (n,%)	17 (17.53)	43 (26.22)	0.128	15 (28.30)	19 (35.85)	0.533
Hypertension (n,%)	75 (77.32)	92 (56.10)	< 0.01	35 (66.04)	31 (58.49)	0.548
Liver Disease (n,%)	10 (10.31)	10 (6.10)	0.216	8 (15.09)	5 (9.43)	0.555
Previous cardiac surgery (n,%)	0 (0)	0 (0)	–	0 (0)	0 (0)	–
Diabetes Mellitus (n,%)	33 (34.02)	58 (35.37)	0.826	16 (30.19)	16 (30.19)	–
Cerebrovascular disease (n,%)	16 (16.49)	22 (13.41)	0.586	9 (16.98)	11 (20.75)	0.804
COPD (n,%)	24 (24.74)	32 (19.51)	0.351	13 (24.53)	16 (30.19)	0.663
Smoking (n,%)	37 (38.14)	50 (30.49)	0.223	21 (39.62)	26 (49.06)	0.434
Alcohol drinking (n,%)	66 (68.04)	103 (62.80)	0.423	35 (66.04)	34 (64.15)	0.839
Preoperative medications						
ACEI/ARB (n,%)	47 (48.45)	57 (34.76)	0.701	18 (33.96)	25 (47.17)	0.235
Ca^2+^-Blocker (n,%)	49 (50.52)	69 (42.07)	0.201	31 (58.49)	22 (41.51)	0.120
β-blocker (n,%)	31 (31.96)	58 (35.37)	0.592	14 (26.42)	18 (33.96)	0.526
Statin (n,%)	27 (27.84)	34 (20.73)	0.226	16 (30.19)	12 (22.64)	0.509
Aspirin (n,%)	97 (100)	164 (100)	–	53 (100.00)	53 (100.00)	–
Diuretics (n,%)	97 (100)	164 (100)	–	53 (100.00)	53 (100.00)	–
Aldosterone antagonist (n,%)	97 (100)	164 (100)	–	53 (100.00)	53 (100.00)	–
Preoperative clinical variables						
detection to surgery (day)	7.45 ± 3.67	8.03 ± 4.02	0.366	7.82 ± 2.89	7.79 ± 3.02	0.892
respiratory support (n,%)	0	0	–	0	0	–
LVEF (%)	20.43 ± 5.29	20.57 ± 4.68	0.824	19.94 ± 5.10	20.66 ± 3.96	0.421
CPB time (minute)	176.93 ± 62.87	182.82 ± 72.61	0.506	178.07 ± 56.37	191.15 ± 72.62	0.303
ACC time (minute)	134.01 ± 47.24	150.06 ± 62.83	0.020	134.23 ± 43.62	128.92 ± 44.75	0.538
Weaning failed (n,%)	34 (35.05)	52 (31.71)	0.579	17 (32.08)	12 (22.64)	0.384
Postoperative VISmax						
VISmax 24H	35.39 ± 7.61	22.79 ± 9.38	0.330	36.06 ± 7.73	23.74 ± 8.19	0.810
VISmax 48H	27.74 ± 5.71	24.55 ± 5.71	0.678	27.80 ± 5.81	26.91 ± 5.81	0.670
VISmax 72H	22.40 ± 3.98	21.28 ± 4.00	0.140	22.25 ± 3.56	22.75 ± 5.92	0.432

*NYHA* New York Heart Association, *LVEF* Left Ventricular Ejection Fraction, *CPB* Cardiopulmonary Bypass, *ACC* Aortic Cross Clamp, *COPD* Chronic obstructive pulmonary disease, *ACEI* Angiotensin-converting-enzyme inhibitor, *ARB* angiotensin receptor blocker, *VIS* Vasoactive Inotropic Score

### Outcomes in the pre-matched patients

**Fig. 2:**
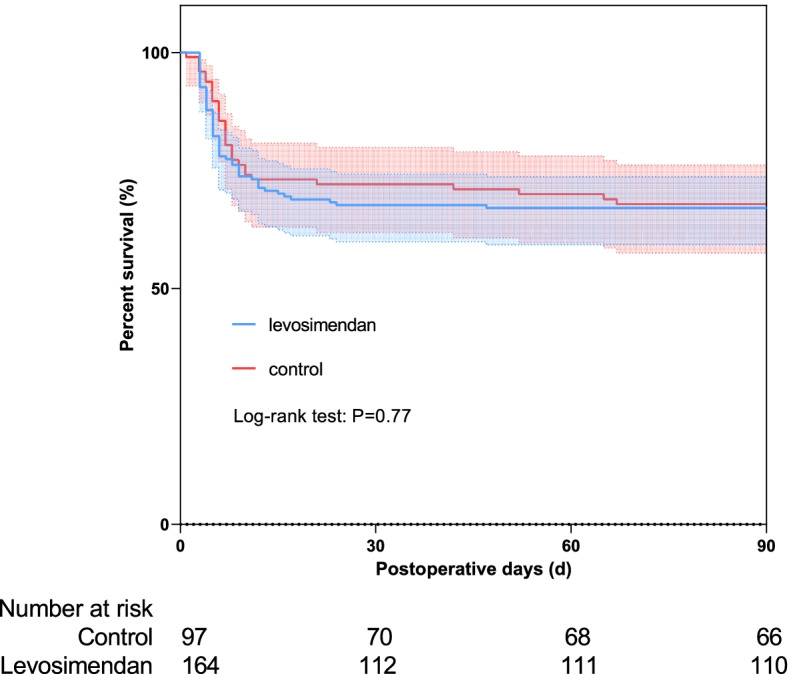
Kaplan-Meier survival curves at 90 days of pre-matched groups. The survival rate was 67.07% for the levosimendan group (blue line), and 68.04% for the control group (red line), *P* = 0.77

After surgical repair, 90-day mortality was 31.96 and 32.93% in the control and levosimendan groups, 30-day mortality were 27.84 and 31.71%, respectively. There was no significant difference in mortality and other outcomes between two groups (Table 2). However, control group had more length of ICU stay (6.69 ± 3.90 d vs. 5.20 ± 2.24 d, *p* < 0.001) and longer MV time (median: 23 h, IQR: 16–53 h vs. median: 16 h, IQR: 11–23 h, *p* < 0.001). The Kaplan–Meier curves and log-rank test of the 90-day survival did not shown significant differences in this pre-matched groups (Fig. 2, *P* = 0.77).

**Table 2:** Postoperative outcomes in pre-matched and propensity-matched patients

Variable	Pre-match			Propensity-match		
	Control (***n*** = 97)	Levosimendon (***n*** = 164)	***P*** value	Control (***n*** = 53)	Levosimendon (***n*** = 53)	***P*** value
Outcoms, (n, %)						
90-day mortality	31 (31.96)	54 (32.93)	0.892	20 (37.74)	16 (30.19)	0.539
30-day mortality	27 (27.84)	52 (31.71)	0.578	17 (32.08)	16 (30.19)	0.834
MCS use						
IABP use	43 (44.33)	60 (36.59)	0.239	27 (50.94)	19 (35.85)	0.170
ECMO use	30 (30.93)	42 (25.61)	0.391	20 (37.74)	12 (22.64)	0.138
Stroke	14 (14.43)	12 (7.32)	0.086	8 (15.09)	5 (9.43)	0.555
AKI by KIDGO (stage III)	43 (44.33)	56 (34.15)	0.114	24 (45.28)	20 (37.74)	0.430
Postoperative Infection, (n, %)	24 (24.74)	39 (23.78)	0.882	11 (20.75)	14 (26.42)	0.648
Septic shock	6 (6.19)	3 (1.83)	0.082	3 (5.66)	0 (0)	0.243
New Atrial fibrillation	4 (4.12)	14 (8.54)	0.213	1 (1.89)	4 (7.55)	0.363
Ventricular arrhythmias	16 (16.49)	42 (25.61)	0.093	10 (18.87)	14 (26.42)	0.487
Length of ICU stay (d)	6.69 ± 3.90	5.20 ± 2.24	< 0.001	6.33 ± 3.22	5.47 ± 2.145	0.111
MV time (h)	23 (16–53)	16 (11–23)	< 0.001	23 (16–53)	18 (13–54)	0.118
Survival time (d)	64.59 ± 37.73	62.90 ± 39.01	0.733	59.57 ± 39.40	64.94 ± 38.53	0.479

Median (Interquartile Range)

Mean ± SD

*MV* Mechanical Ventilation, *ECMO* Extracorporeal Membrane Oxygenation, *AKI* Acute Kidney Injury, *KIDGO* Kidney Disease Disease Improving Global Guidelines, *MCS* Mechanical circulatory support

### Outcomes in the propensity-matched patients

**Fig. 3:**
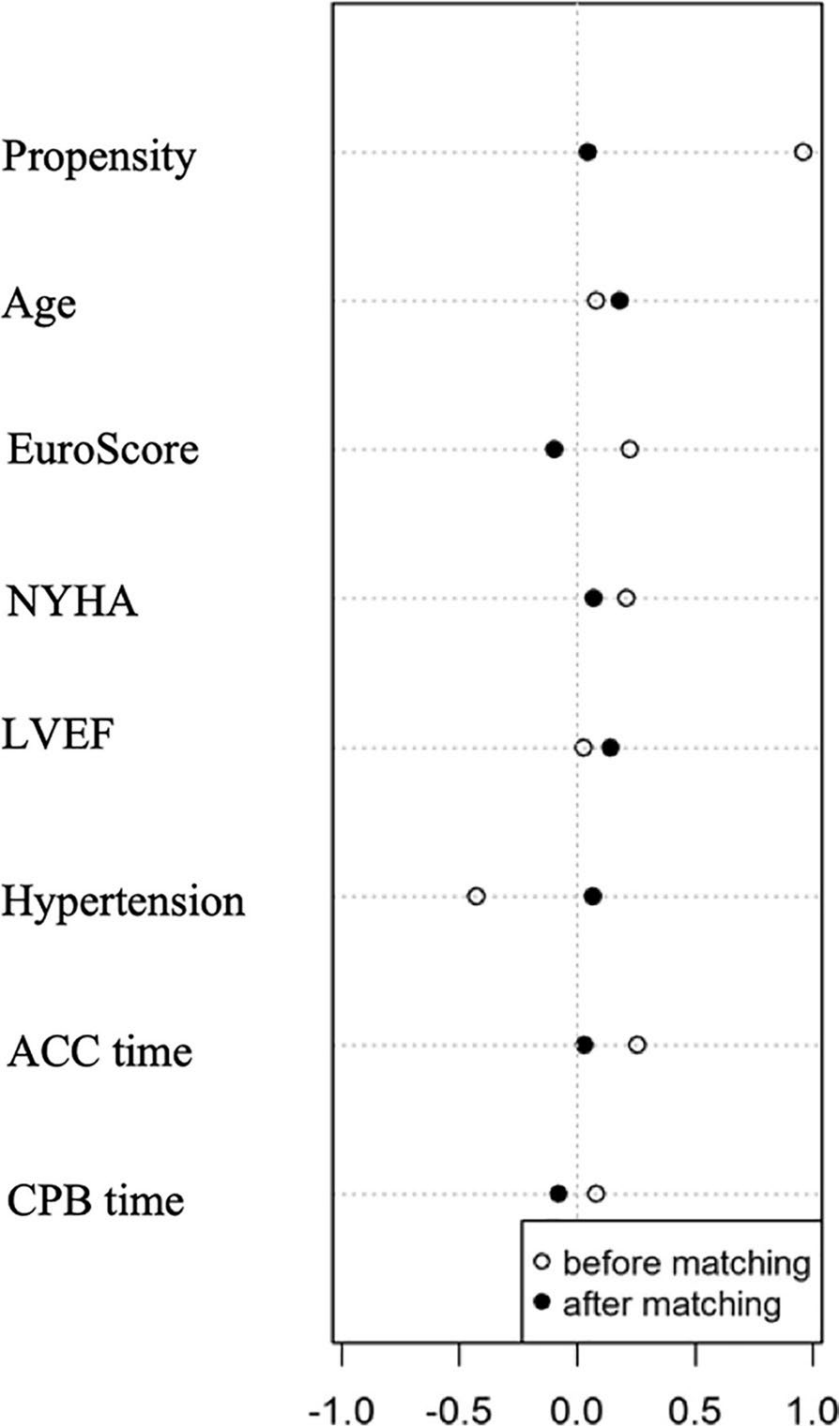
Standardized Mean Difference (SMD) of clinical variables after match

**Fig. 4:**
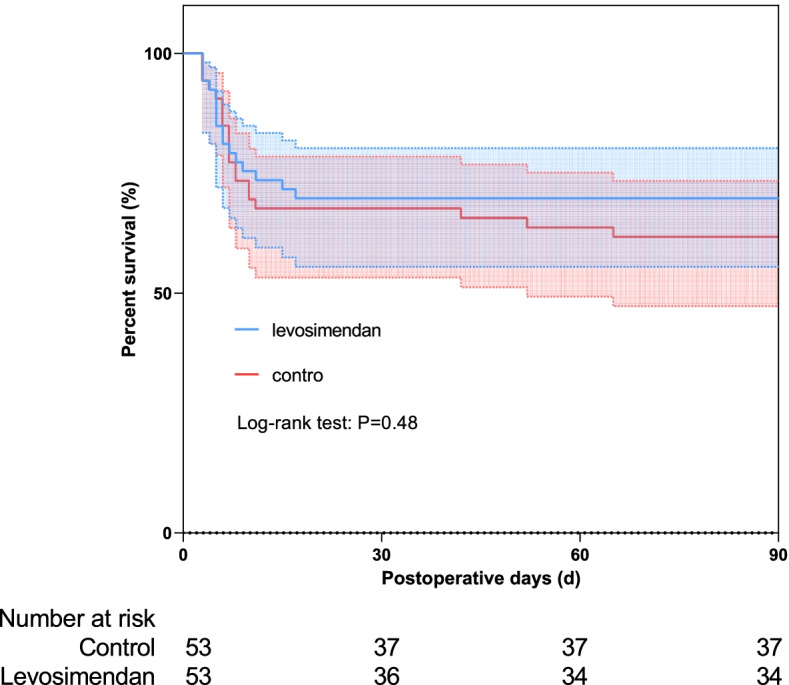
Kaplan-Meier survival curves at 90 days of propensity-matched groups. The survival rate was 69.81% for the levosimendan group (blue line), and 62.26% for the control group (red line), *P* = 0.48

We used propensity score analysis for further assessment. Levosimendan administration was associated with multiple clinical variables, including age, EuroSCORE, NYHA class, hypertension Left Ventricular Ejection Fraction (LVEF), Aortic Cross Clamp (ACC) time and cardiopulmonary bypass time. Finally, there were 106 patients (53 levosimendan and 53 control) in the propensity-matched cohort. Standardized Mean Difference (SMD) was used for the assessment of balance after match, and all the SMD of clinical variables was < 0.1 (Fig. 3). The baseline characteristics among propensity-matched groups was no significant difference (Table 1). No significant difference was found between both groups for all outcomes and complications. The Kaplan–Meier curves and log-rank test of the 90-day survival did not shown significant differences in matched groups (Fig. 4, *P* = 0.48).

## Discussion

In our study, we focused on patients undergoing VSR repair with CABG. Our results were shown as follows: ① The mortality, AKI, MCS use and stroke had no differences significantly between the levosimendan and control group. Before matching, the levosimendan group might decrease length of ICU stay and mechanical ventilation time compared with control group; ② In order to eliminated potential bias, we used propensity score analysis for further assessment. After matching, all postoperative outcomes, including length of ICU stay and mechanical ventilation time, were not different among two groups; ③The Kaplan–Meier curves showed 90- days survival had no differences among two groups before and after matching.

Levosimendan had been identified as a helpful agent to decrease mortality in patients undergoing cardiac surgery [15, 16, 29]. However, there were some contrary results that had been reported in recent studies [18–20]. It is still controversial whether levosimendan had positive effects or not on patients undergoing CABG. And to our best knowledge, there was no study that had been focused on AMI-VSR. We, therefore, designed this study to evaluate the efficacy of levosimendan in patients with VSR underwent cardiac surgery. In our study, levosimendan did not reduce the 90-day mortality. Moreover, no benefit of levosimendan was found on postoperative complications.

Series of experimental studies reported that levosimendan was an activator of potassium ATPase channel which improved potassium flux to the mitochondrial matrix [30], and modulated mitochondrial ATP production and implicated a pharmacological mechanism for cardioprotection [31]. Additionally, levosimendan also as a Ca2+ sensitizer to increase Ca2 + −saturated cardiac troponin C in cardiomyocytes [32], which improved cardiac performance without oxygen wasting [33, 34]. Theoretically, the levosimendan may be beneficial in patients with ischemic cardiomyopathy and heart failure. Then, some clinical studies were managed to investigate the efficacy of levosimendan. Erikkson et al. [35] reported that levosimendan significantly enhanced primary weaning from CPB and decreased IABP use in on-pump coronary artery bypass grafting (op-CABG). De Hert et al. [36] reported that levosimendan could significantly improve ventricular function in patients with low preoperative LVEF. Then, three large multicenter randomized trials were following developed to supply high-quality evidence. However, they found that levosimendan had no benefit for patients with pre-operative left ventricular (LV) dysfunction. In the LEVO-CTS trial, it demonstrated that levosimendan did not show the superiority in the poor outcomes in patients with LVEF < 35% compared with placebo [19]. In the CHEETAH trial, 30-day mortality had no differences between levosimendan and placebo in patients with severely perioperative LV dysfunction [37]. The LICORN trial reported that there were no any clinical advantages in patients with LVEF≤40% underwent cardiac surgery [20]. However, some recent researches reposted that levosimendan was associated with lower 90-day mortality or LCOS in patients underwent isolated CABG, but not in those underwent other procedures [38–40]. It may be necessary to evaluate the levosimendan by a single disease. However, the previous studies did not report what effects of levosimendan on AMI-VSR patients.

Our study failed to demonstrate any advantage in levosimendan supported patients with VSR. However, there are still many issues that deserve further consideration. First, for patients who has developed VSR after AMI and undergoing surgical repair, the operative mortality has not been improved significantly over the past half century [4]. This trend may suggested that advances in surgical technology may not be beneficial for such patients. The theoretical basis of levosimendan for the treatment of ischemic cardiomyopathy was still solid, at the same time we are in absence of more and new options with inotropic agents, in fact, levosimendan represents a rare case of an inotrope for short-term hemodynamic treatments for acute cardiac care which approved by regulatory authorities in the past 20 years. Some studies showed that levosimendan was a valid pharmacological strategy for perioperative management of VSR [41, 42]. This result prompts optimal use timing of levosimendan need more exploration. Then, we can not deny the possibility that higher doses levosimendan might have been effective in reducing mortality and complications, although higher doses might also have increased the risk of hypotension and arrhythmias. Third, it is hard to known about actual viable myocardium in patients between two groups and we did not systematically collect cardiac output data, which can lead to the real efficacy of levosimmendan being masked.

In summary, our study found that a low-dose infusion of levosimendan in VSR patients underwent surgical repair and CABG did not associated with lower mortality and not associated with positively affect to postoperative outcomes.

### Study limitation

Our study was two centers prospective observational cohort study. Some potential biases could have been influenced this study and we used propensity score matching to avoid them. In another side, some hard to be observed factors but actually affect assignment to treatment and outcomes could have been lost in the matching procedure. Hidden bias attribute to latent variables might still remain after matching, which may cause statistical errors. Furthermore, propensity score matching removed a large number of patients may lead to an increase in statistical error. In addition, the condition of AMI-VSR was very complicated, the recruited patients in our study might not reflect well to the clinical reality. This study could not cover every part of hemodynamic situations. It may cause some potential errors.

## Data Availability

The datasets generated and/or analysed during the current study are not publicly available due to patients did not signed consents about upload the data but are available from the corresponding author on reasonable request.
